# The Female Athlete’s Heart: Facts and Fallacies

**DOI:** 10.1007/s11936-018-0699-7

**Published:** 2018-11-03

**Authors:** Clea Simone S. S. Colombo, Gherardo Finocchiaro

**Affiliations:** 1 MSc Sports Cardiology, Cardiology Clinical Academic Group, St George’s University of London, Cranmer Terrace, SW 17 0RE, London, UK; 2 Cuore Dello Sport, Valinhos, R. Luiz Spiandorelli Neto, 60, s307. Valinhos, São Paulo, Brazil; 3 Cardiology Clinical Academic Group, St George’s University of London, Cranmer Terrace, SW 17 0RE, London, UK

**Keywords:** Female athletes, Cardiac remodeling, Sudden cardiac death

## Abstract

**Purpose of the review:**

For many years, competitive sport has been dominated by men. Recent times have witnessed a significant increase in women participating in elite sports. As most studies investigated male athletes, with few reports on female counterparts, it is crucial to have a better understanding on physiological cardiac adaptation to exercise in female athletes, to distinguish normal phenotypes from potentially fatal cardiac diseases. This review reports on cardiac adaptation to exercise in females.

**Recent findings:**

Recent studies show that electrical, structural, and functional cardiac changes due to physiological adaptation to exercise differ in male and female athletes. Women tend to exhibit eccentric hypertrophy, and while concentric hypertrophy or concentric remodeling may be a normal finding in male athletes, it should be evaluated carefully in female athletes as it may be a sign of pathology. Although few studies on veteran female athletes are available, women seem to be affected by atrial fibrillation, coronary atherosclerosis, and myocardial fibrosis less than male counterparts.

**Summary:**

Males and females exhibit many biological, anatomical, and hormonal differences, and cardiac adaptation to exercise is no exception. The increasing participation of women in sports should stimulate the scientific community to develop large, longitudinal studies aimed at a better understanding of cardiac adaptation to exercise in female athletes.

## Introduction

Regular intensive exercise leads to a series of electrical, structural, and functional changes in the heart, collectively named as the “athlete’s heart” [1]. Most of the studies on cardiac adaptation to exercise have investigated male athletes. Until the first half of the twentieth century, women were discouraged from practicing sport at a competitive level because females were thought to be physiologically unable to perform at high standards, and it was believed that intense exercise could lead to female infertility [2]. Women’s participation in competitive sports has progressively increased since the 1980s, and the proportion of females among marathon runners has increased from 10% in 1980 to 43% in 2013 [3]. Females comprised almost half of the athletes participating in the Olympic Games in Rio de Janeiro in 2016 [4].

Although the recent years have witnessed an increasing participation of women in competitive sport activities, data on cardiac adaptation in female athletes are limited. Recent studies showed important gender differences in patterns of electrical and structural cardiac adaptations [5•, 6••]. Incidence of sudden cardiac death (SCD) in sports is significantly lower in female athletes (male/female ratio is 10:1), and it is possible that gender-related differences in cardiac adaptation to exercise may offer some explanation [7].

## The athlete’s heart

Regular intense exercise training promotes electrical, structural, and functional cardiac changes which are influenced by age, sex, body size, ethnicity, and sport discipline [8]. Physiological cardiac adaptation usually results in symmetrical enlargement of all cardiac chambers and increased vagal tone, with typical ECG changes as sinus bradycardia, AV blocks, and voltage criteria for right and left ventricular hypertrophy. Early repolarization is commonly observed in athletes and anterior T wave inversion (TWI) may be found, especially in individuals of Afro-Caribbean descent. Cardiac remodeling may be marked in some athletes, resulting to significant overlap with potentially fatal cardiac disease such as cardiomyopathies [1].

Highly trained athletes exhibit an increase in left and right ventricular size of 10–15% when compared with sedentary individuals. In Olympic athletes, left ventricular (LV) size has been shown to be higher than the upper limits of normal (LV end-diastolic diameter > 54 mm) in almost half of the cases. In some athletes, especially those engaged in high dynamic modalities, such as rowing, cross-country, and cycling, the LV end-diastolic diameter may exceed 60 mm [9]. The LV wall thickness may be mildly increased in white athletes, but rarely exceeds 12 mm [10] whereas athletes of Afro-Caribbean descent exhibit increased wall thickness (> 12 mm) in up to 18% of the cases [11]. Although LV systolic function is usually within conventional normal limits in athletes, endurance athletes may exhibit mildly reduced LV ejection fraction (< 50%) at rest, with significant contractility recruitment during exercise [12, 13].

Similar to the LV, the right ventricle (RV) also enlarges in athletes. Zaidi et al. showed that at echocardiography, 61% of male and 46% of female athletes exhibit RV dimensions that fulfill the minor diagnostic criterion for arrhythmogenic right ventricular cardiomyopathy (ARVC) [14].

## Cardiac adaptation to exercise in female athletes

Although cardiac physiological adaptation may be qualitatively similar in male and female athletes, on average women are smaller, have lower lean body mass, and a different hormonal profile than men with significant impact on cardiac dimensions [15]. In addition, sympathetic adrenergic response during exercise as well as peak systolic blood pressure, stroke volume, and peak VO2 during exercise may be lower in female athletes [16, 17] . These differences may impact significantly on cardiac structural changes resulting from intense training.

### Electrocardiogram

**Fig. 1:**
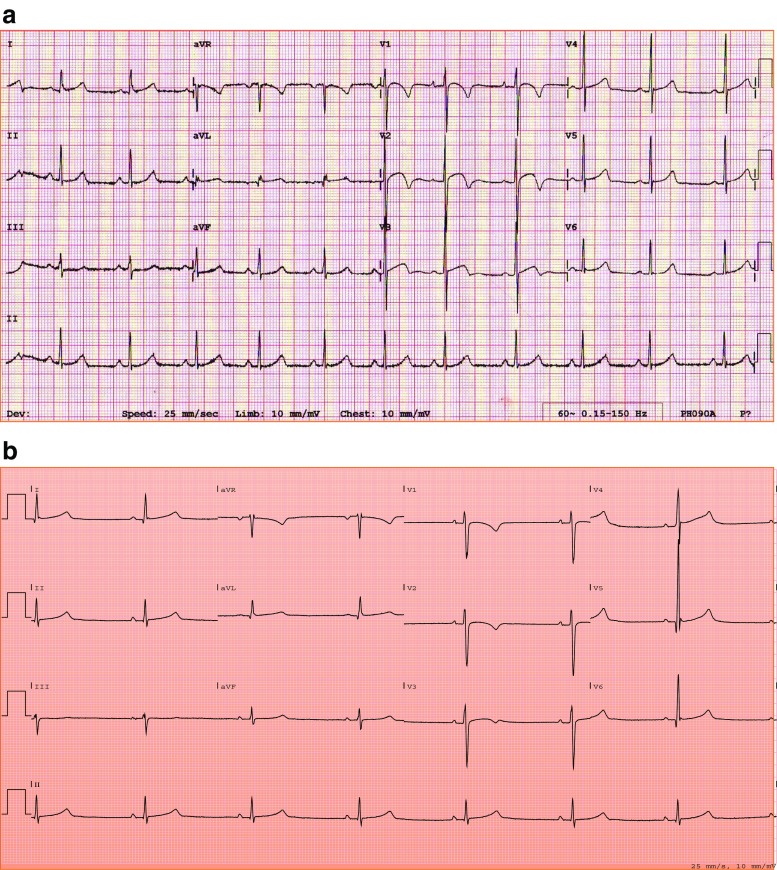
ECG patterns in male (**a**) and female (**b**) athletes. Note the combination of anterior T wave inversion and significant (> 0.1 mV) J point elevation and the voltage criteria for LVH in the male athlete’s ECG. Conversely, the female athlete’s ECG is characterized by the absence of J point elevation and voltage criteria for LVH.

Highly trained athletes often exhibit ECG patterns, which are reflective of physiological adaptations to exercise and may in some cases overlap with conditions predisposing to sudden cardiac death (SCD). The differential diagnosis between physiology and pathology may be challenging, and therefore, a good understanding of what constitutes a normal ECG in specific athletic sub-groups is necessary. The recently published International recommendations for ECG interpretation in athletes provide a thorough guidance for the interpretation of the athlete’s ECG, specifying what type of ECG abnormalities would require further investigations to rule out pathology [18•]. However, this document is predominantly based on studies of male athletes. Recent studies show that sex has a significant effect on ECG patterns in athletes. Finocchiaro et al. [6••] showed that Sokolow-Lyon (SL) voltage criteria for left ventricular hypertrophy (LVH) as well as left axis deviation are less common in female than in male elite athletes in a study including more than 1000 highly trained athletes. The SL criteria for LVH were present in 14% of females compared with 42% of males, while left axis deviation was present in 0.4% of females compared with 4% of males. Conversely, anterior T wave inversion (TWI) was more prevalent in female athletes (9% in females vs 4% in males) [5•, 6••]. These findings were confirmed by the study by Malhotra et al. [5•], where anterior TWI were found in 6.5% of female athletes and in 2.1% of male athletes. The combination of J point elevation > 0.1 mV in V1-V2 and anterior TWI may be observed in male athletes, especially of Afro-Caribbean origin, and this feature is regarded as a sign of physiological adaptation to exercise [19]. However, this ECG sign is rarely observed in highly trained female athletes, and its absence should not be considered as suggestive of pathology [20] (Fig. 1).

On the largest study on black female athletes, Rawlins et al. [21] showed that female athletes of Afro-Caribbean descent exhibit more frequently anterior TWI than white counterparts.

### Echocardiogram

In male athletes, cardiac adaptation to exercise results in structural remodeling of both ventricles and atria. As body size differs between males and females, absolute cardiac dimensions are lower in females reflecting the different anthropometry [6••]. Scaling cardiac dimensions for body size is particularly relevant in this context, in order to have a more accurate appreciation of the degree of cardiac remodeling.

#### Left ventricle

On a large cohort (*n* = 600) of elite female athletes, Pelliccia et al. [22] showed that an LV end-diastolic diameter (LVEDD) > 54 mm, consistent with a diagnosis of dilated cardiomyopathy (DCM), was present in 8% of athletes. Maximal LV wall thickness was 14% greater in female athletes in comparison with sedentary counterparts, but 23% lower than male athletes. Importantly, none of the female athletes showed a LV wall thickness > 12 mm [22]. Therefore, an enlarged left ventricle raising the suspicion of DCM or a wall thickness consistent with hypertrophic cardiomyopathy (HCM) is less common in female athletes than male counterparts. A female athlete showing an LV cavity exceeding 60 mm or a LV wall thickness > 12 mm should be further investigated to exclude cardiac pathology. More recently, Finocchiaro et al. [6••] described the effect of sex and sport discipline on LV geometry in 1083 white elite athletes (41% female athletes). Absolute LVEDD > 54 mm was observed only in 7% of female athletes and LV wall thickness was ≤ 12 mm in all females, confirming previous studies. Interestingly, LVEDD indexed for BSA was higher in female athletes (LVEDD ≥ 31 mm/m^2^ in 18% of females vs 10% of males). In the specific subgroup of athletes engaged in dynamic exercise, females showed eccentric hypertrophy more frequently than males, while males showed more commonly concentric remodeling/hypertrophy. None of the female athletes demonstrated RWT > 0.48 or LVM > 145 g/m^2^ [6••].

**Fig. 2:**
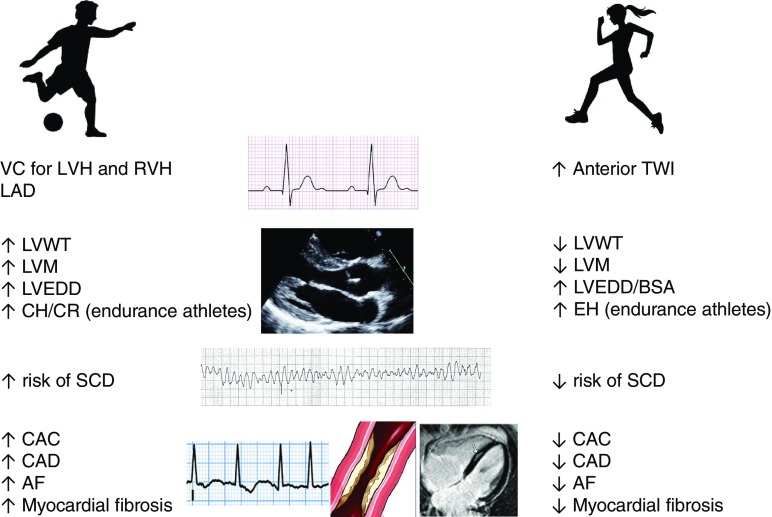
Main gender differences in cardiac adaptation to exercise**.** Abbreviations: AF: atrial fibrillation; BSA: body surface area; CAC: coronary artery calcification; CAD: coronary artery disease; CH: concentric hypertrophy; CR: concentric remodeling; EH: eccentric hypertrophy; LAD: left atrial dilation; LVEDD: left ventricular end diastolic diameter; LVH: left ventricular hypertrophy; LVM: left ventricular mass; LVWT: left ventricular wall thickness; RVH: right ventricular hypertrophy; VC: voltage criteria.

Rawlins et al. [21] compared 200 black female athletes with white female athletes of similar age, size, and type of sport and demonstrated that black female athletes exhibited a LV wall thickness > 11 mm in 3% of the cases compared with none of white female athletes. No female athlete exhibited a LV wall thickness > 13 mm [21]. These findings (Table 1 and Fig. 2) suggest that cardiac adaptation to exercise tends to be characterized by a more significant increase in chamber size rather than in wall thickness in females. The presence of concentric hypertrophy or concentric remodeling in female athletes should be evaluated carefully and may be suggestive of HCM in individuals with a family history of premature disease or with an abnormal ECG.

**Table 1:** Chamber dimensions at echocardiography in female athletes

**Left ventricle**			
	**Pelliccia et al. 1996**	**Rawlins et al. 2010**	**Finocchiaro et al. 2016**
Number ethnicity	600 females White	200 females Black	438 females White
LVWT > 12 mm	0%	2%	0%
LVEDD > 54 mm	8%	8%	7%
LVEDD index	29.8 ± 2.5 mm/m^2^	–	28.6 ± 2.7 mm/m^2^
LVM index	80 ± 16 g/m^2^	–	83 ± 17 g/m^2^
**Left atrium**			
	**Pelliccia et al. 1996**	**Rawlins et al. 2010**	**Iskandar et al. 2015**
Number ethnicity	600 females White	200 females Black	7018 athletes mixed
LA diameter	32.5 ± 3.5 mm	35.3 ± 4.7 mm	34.2 mm
LA volume index	–	–	30.9 ml/m^2^
**Right ventricle and right atrium**			
	**D’Ascenzi et al. 2017**	**Zaidi et al. 2013**	**D’Ascenzi et al. 2017**
Number ethnicity	363 females White	57 females Black	6806 athletes mixed
RVOTP	26.1 ± 3.6 mm	26.7 ± 3.8 mm	28 ± 2.0 mm
RVOT1	27.3 ± 4.1 mm	28.1 ± 4.5 mm	30 ± 1.0 mm
RVEDA	19.3 ± 3.9 cm^2^	21.6 ± 3.8 cm^2^	23 ± 0.1 cm^2^
RA area	14.8 ± 3.4 cm^2^	14.4 ± 3.1 cm^2^	16 ± 1.0 cm^2^

*LA* left atria, *LVEDD* left ventricular diastolic diameter, *LVM* left ventricular mass, *LVWT* left ventricular wall thickness, *RA* right atria, *RVEDA* right ventricular end diastolic area, *RVOT* right ventricular outflow tract, *RVOTP* right ventricular outflow tract parasternal

#### Right ventricle

Exercise-induced cardiac remodeling is not a prerogative of the left heart and the right heart is known to be very sensitive to volume loading conditions, especially in endurance athletes. Zaidi et al. [14] showed that although absolute RV dimensions are larger in male athletes compared with females, RV size indexed for BSA is higher in female than male athletes [23, 24]. This is particularly relevant as RV enlargement may overlap with the pathological dilatation of the RV in patients with ARVC. Athletic training has several deleterious effects in terms of risk of arrhythmias and phenotype progression in patients with ARVC or even in carriers of desmosomal mutations, and therefore, the differential diagnosis is particularly relevant in athletes [25–27]. Echocardiographic evaluation of the RV should not rely on size alone, but also on other features such as the presence of regional wall motion abnormalities or possible aneurysms in order to distinguish RV alterations typical of ARVC from those detected in athletes as a consequence of intensive physical activity [28, 29].

#### Atria

Left and right atrial enlargements are often observed in highly trained athletes. In a recent meta-analysis of 54 studies, Iskandar et al. [30] confirmed that athletes had greater LA size in comparison with controls with a 13% increase in LA diameter and a 30% increase in LA volume index. The mean LA diameter was 36 mm in male elite athletes and 34 mm in female elite athletes, and the mean diameter was 4.1 mm greater in comparison with sedentary controls [30]. Right atrial remodeling is particularly evident in endurance athletes, as a response to the prolonged increase in cardiac output [31–33]. Although most studies included male athletes, in order to standardize right cardiac measurements, Zaidi et al. [14] proposed an upper limit for RA area of 28 cm^2^ in male athletes and 24 cm^2^ in female athletes. Interestingly, these authors found no significant differences between black and white male athletes but greater RA dimensions in white female athletes.

## Determinants of cardiac adaptation to exercise in female athletes

The determinants of sex differences in physiological cardiac adaptation to exercise are not fully understood. However, hormonal, molecular, and genetic mechanisms are thought to be involved.

### Hormonal mechanisms

Testosterone and its metabolite dihydrotestosterone act directly on cardiac myocytes stimulating myocardial hypertrophy [34, 35]. Conversely, estrogens inhibit myocardial hypertrophy and the proliferation of cardiac fibroblasts. The myocardial response to these hormones does not solely depend on the circulating hormonal levels but also on their binding to cardiac receptors. Myocytes contain androgenic and estrogenic receptors, which seem to modulate the expression of specific cardiac genes with possible effects on sarcomeric proteins [36, 37]. Moreover, estrogens increase nitric oxide level promoting peripheral vasodilatation and afterload reduction, and estrogens and testosterone have opposite effects on circulating levels of angiotensin converting enzyme activity promoting, respectively, peripheral vasodilation and vasoconstriction with different effects on blood pressure [38–40].

### Molecular mechanisms

Cardiac remodeling is modulated by multiple signaling pathways which involve enzymatic regulation and cellular response. The energy substrate availability which depends on fatty acid oxidation and glucose uptake seems to influence physiological cardiac hypertrophy. Some enzymes involved in this process, as protein kinase B (AKT) and glycogen synthase kinase-3-beta (GSK 3), prevent the development of cardiac hypertrophy and their activity is reported to be higher in females [41, 42]. Although these findings derive mainly from experimental models, with lack of data in humans, they could explain, at least in part, sex differences in cardiac remodeling.

### Genetic mechanisms

A significant variability characterizes genes encoding for protein components of the renin-angiotensin system [43]. Studies on athletes showed a significant association between specific polymorphisms in the gene encoding for angiotensin and degree of LVH induced by endurance training. Sex is supposed to have an important role on the prevalence and expression of these polymorphisms possibly explaining the differences in cardiac adaptation to exercise [44, 45].

## Sudden cardiac death in female athletes

Sudden cardiac death (SCD) is ten-fold less common among competitive female athletes in comparison with male counterparts [46, 47].

The commonest cause of SCD in older athletes (> 35 years) is atherosclerotic coronary artery disease (CAD), whereas in young athletes (< 35 years), inherited cardiac diseases, such as cardiomyopathies and channelopathies, predominate [46]. Recent studies suggest that the most common finding at autopsy in young athletes who died suddenly is a structurally normal heart (sudden arrhythmic death syndrome or SADS), where primary arrhythmia syndromes as long QT syndrome, Brugada syndrome, and cathecolaminergic polymorphic ventricular tachycardia (CPVT) are the probable culprits [48, 49].

The higher prevalence of SCD in males was previously explained with the higher proportion of male athletes participating in competitive sports. However, the increasing participation of women in the recent years was not paralleled with an increase of SCD incidence in female athletes.

Various theories to explain the higher incidence of SCD in male athletes have been proposed. Sympathetic tone and increased catecholamines may trigger malignant arrhythmias in individuals with pre-existing cardiac disease and males appear to have a higher sympathetic tone than females with possible deleterious effects in predisposed individuals [16, 50, 51].

Sex hormones affect the regulation of cardiac repolarization, with effects on the QT interval with possible development of electrical instability and arrhythmias [52, 53]. While androgens may have a role in the development of LVH, estrogens may delay the phenotype expression in HCM, which may manifest later in women [54–56], and although both HCM and ARVC are transmitted as Mendelian autosomal dominant trait, their prevalence is higher in males [28, 54]. The interplay between genetic and environmental factors in the development of cardiomyopathies, which are important causes of SCD especially in young athletes, is complex and not completely understood.

## Veteran female athletes

Regular exercise has several beneficial effects for human health, decreases morbidity, and improves survival. Increasing numbers of athletes are able to continue their athletic activities into middle and even old age, participating at high-level athletic activity for many decades. Recent studies postulated a deleterious effects of high-intensity long-term exercise, which has been associated with greater prevalence of atrial fibrillation (AF), ventricular arrhythmias, coronary artery calcification (CAC), and myocardial fibrosis in veteran athletes [57, 58••]. Most of these studies investigated the effects of long-term training in veteran male athletes, and to date, few studies included female athletes.

### Atrial fibrillation

A higher prevalence of atrial fibrillation (AF) in athletes compared with sedentary counterparts has been recently demonstrated [59]. A recent meta-analysis on the topic reported a prevalence of male athletes in studies where an association between AF and sport was demonstrated [60].

The Women’s Health Study, which enrolled middle-age healthy women, showed that while a moderate amount of physical activity is associated with lower prevalence of AF, vigorous exercise regimens may increase this risk of developing AF [61]. However, female athletes exhibit a less pronounced atrial remodeling than male athletes, and larger atria are more susceptible to micro re-entries favored by the increase in adrenergic tone suggesting a possible lower risk of developing AF in women [62]. Although data on large cohort of male athletes suggest a 5-fold risk of developing AF, studies on female athletes are on small samples, leaving questions on the prevalence of AF in this specific group of individuals still un-answered.

### Coronary artery disease

A possible role of long-term strenuous exercise in the development of coronary atherosclerosis has been recently shown in veteran athletes [63–65]. A study comparing veteran endurance athletes with low atherosclerotic risk profile and sedentary controls showed a higher prevalence of significant coronary plaques and higher values of CAC in male athletes [58••]. Female athletes constituted only 30% of the overall population, and the prevalence of CAD or CAC score was similar in comparison with female controls. A comparison between sedentary middle-age women and women of similar age engaging in marathon running demonstrated a lower prevalence of coronary plaques and less calcified plaques in athletes, suggesting that the possible effects of long-term intense athletic training on coronary atherosclerosis may be different in men and women [66•]. Further studies on larger cohorts are needed to clarify if female veteran athletes are less likely to develop coronary atherosclerosis.

### Myocardial fibrosis

Recent studies using cardiac magnetic resonance (CMR) showed a higher prevalence of late gadolinium enhancement (LGE) in marathon and ultra-marathon runners compared with age-matched veteran controls [67, 68]. A correlation between the duration of training or number of marathons completed and the presence of myocardial fibrosis was reported. It is possible that repeated bouts of myocardial inflammation due to intense exercise lead to a permanent damage, which may result in arrhythmias [69, 70]. The LGE pattern is usually non-ischemic indicating that myocardial fibrosis is not related to CAD.

Again, most of the studies have included male athletes. A recent study reported on male and female triathletes who were investigated with CMR showing that LGE was present in 17% of males, but in none of the females [71]. Interestingly, athletes showing LGE were characterized by higher systolic blood pressure at peak exercise and increased LV mass index than athletes without LGE at CMR, and the systolic blood pressure at peak exercise was independently associated with myocardial fibrosis. It is possible that, as female athletes exhibit lower values of blood pressure at rest and during exercise, they are less prone to develop myocardial fibrosis. Myocardial fibrosis may constitute a substrate for potentially fatal arrhythmias and SCD, and its lower prevalence in female athletes may explain the lower incidence of SCD.

## Conclusion and future perspectives

Long-term training results in a plethora of electrical, structural, and functional cardiac changes. Patterns of electrical and structural remodeling differ significantly between male and female athletes. While male athletes tend to exhibit more frequently voltage criteria for LVH and RVH at the ECG, in female athletes, anterior TWI is more common. Absolute cardiac dimensions are higher in male athletes, and female athletes rarely exhibit an enlarged LV raising the suspicion of DCM or LV wall thickness consistent with HCM. Endurance female athletes appear to adapt primarily by increasing ventricular dimensions as opposed to an increase in LV wall thickness. Finally, female athletes are less prone to sudden cardiac death and show less frequently coronary calcification, atrial fibrillation, and myocardial fibrosis in comparison with male counterparts.

Undoubtedly, males and females exhibit many biological, anatomical, and hormonal differences, and cardiac adaptation to exercise is no exception. The increasing participation of women in sports should stimulate the scientific community to develop large and longitudinal studies aimed at a better understanding of cardiac adaptation to exercise in female athletes.

### What we know about…

#### ECG findings

- Anterior TWI is more common in female than male athletes.
- Voltage criteria for LVH and RVH are less frequent in female athletes.

#### Echocardiogram findings

- Female endurance athletes tend to develop eccentric hypertrophy.
- LVH > 13 mm, RWT > 0.48, or LVM > 145 g/m^2^ in female athletes is suggestive of pathology.
- RV enlargement is common in female athletes.

#### Clinical evolution

- Veteran female athletes show less coronary artery calcification than males.
- Female athletes are less prone to SCD than males.

### What we believe…

- Sex hormones and genetic factors play a role in cardiac remodeling.
- A better understanding of the mechanisms involved in cardiac adaptation to exercise in males and females may be useful to prevent SCD.

## Abbreviations

AF: atrial fibrillation
ARVC: arrhythmogenic right ventricular cardiomyopathy
CAC: coronary artery calcification
CAD: coronary artery disease
CMR: cardiac magnetic resonance
DHT: dihydrotestosterone
HCM: hypertrophic cardiomyopathy
LV: left ventricular
LVEDD: left ventricular end diastolic diameter
LVH: left ventricular hypertrophy
LVM: left ventricular mass
LVWT: left ventricular wall thickness
RV: right ventricle
RWT: relative wall thickness
SCD: sudden cardiac death
TWI: T wave inversion
